# SPECT and PET Imaging of Meningiomas

**DOI:** 10.1100/2012/412580

**Published:** 2012-05-01

**Authors:** Varvara Valotassiou, Anastasia Leondi, George Angelidis, Dimitrios Psimadas, Panagiotis Georgoulias

**Affiliations:** ^1^Nuclear Medicine Department, University Hospital of Larissa, Mezourlo, 41110 Larissa, Greece; ^2^Nuclear Medicine Department, “Alexandra” University Hospital, Vas. Sofias 80, 11528 Athens, Greece; ^3^Nuclear Medicine Department, NIMTS Hospital, Monis Petraki 10-12, 11521 Athens, Greece

## Abstract

Meningiomas arise from the meningothelial cells of the arachnoid membranes. They are the most common primary intracranial neoplasms and represent about 20% of all intracranial tumors. They are usually diagnosed after the third decade of life and they are more frequent in women than in men. According to the World Health Organization (WHO) criteria, meningiomas can be classified into grade I meningiomas, which are benign, grade II (atypical) and grade III (anaplastic) meningiomas, which have a much more aggressive clinical behaviour. Computed Tomography (CT) and Magnetic Resonance Imaging (MRI) are routinely used in the diagnostic workup of patients with meningiomas. Molecular Nuclear Medicine Imaging with Single Photon Emission Computed Tomography (SPECT) and Positron Emission Tomography (PET) could provide complementary information to CT and MRI. Various SPECT and PET tracers may provide information about cellular processes and biological characteristics of meningiomas. Therefore, SPECT and PET imaging could be used for the preoperative noninvasive diagnosis and differential diagnosis of meningiomas, prediction of tumor grade and tumor recurrence, response to treatment, target volume delineation for radiation therapy planning, and distinction between residual or recurrent tumour from scar tissue.

## 1. Introduction

Meningiomas arise from the meningothelial cells of the arachnoid membranes, which are attached to the inner layer of the dura mater [1]. They are the most frequently reported primary intracranial neoplasms representing about 20% of all intracranial tumors [2]. They are usually diagnosed after the third decade of life and they are more frequent in women than in men accounting for 38% of all intracranial tumors in women and 20% in men [3].

Meningiomas can be differentiated histologically into 15 subtypes [2] and classified into three grades (I–III) according to the World Health Organization (WHO) criteria, which have been updated in 2007 [4, 5]: grade I meningiomas which are benign, exhibit slow growth, and represent 90%; grade II (atypical) and grade III (anaplastic) meningiomas, representing about 6–8% and 2–4%, respectively, which have a much more aggressive clinical behaviour and they are characterized by rapid progression, recurrence, metastases, and poor outcome [2]. More specifically, atypical meningiomas have high mitotic index and presence of at least three of the following four characteristics: sheeting architecture, hypercellularity, macronuclei, and small cell formation. Anaplastic meningiomas are characterized by excessive mitotic activity and focal or diffuse loss of meningothelial differentiation at the light microscopic level resulting in sarcoma, carcinoma or melanoma-like appearance [5].

Brain invasion is a controversial criterion. Although it is not formally a pathologic criterion for WHO grade II meningiomas; nevertheless, the diagnosis should mention it since the brain invasion corresponds prognostically to WHO grade II and its presence implies a greater likelihood of recurrence [1].

Surgical resection of meningiomas is the prevalent treatment. Moreover, radiation therapy has now attained a standard role in the primary and adjuvant treatment settings and is indeed the only commonly accepted form of adjuvant therapy after surgical resection, although meningiomas have, errantly, been regarded as radio resistant [6].

The risk of recurrence in operated meningiomas is correlated with the histological grade, the degree of surgical resection, the biological aggressiveness of the tumor, and large tumor size [7]. Although in benign meningiomas surgical resection is associated with a high cure rate, supposing that the whole tumor is excised; however, there is always considerable risk of recurrence (ranged between 9% and 32%) even after apparently complete resection with excision of the surrounding dura and involved bone [8, 9]. On the contrary, recurrence is very common in high-grade lesions, even after total resection [10]. Biological aggressiveness of meningiomas depends on various cellular characteristics such as the proliferation activity, which is determined by the expression of the nuclear antigen Ki-67 [11].

In the diagnostic workup of patients with meningiomas, a combination of contrast-enhanced imaging techniques such as Computed Tomography (CT) and Magnetic Resonance Imaging (MRI) is routinely used in defining the location and extent of tumour, as well as for long-term followup. Although these imaging modalities could provide a presumptive diagnosis, the histological diagnosis can only be made postoperatively. Furthermore, this combination has also limitations, especially at the skull base and in the case of bony involvement, while in the case of suspected residual or recurrent tumour, it can be very difficult to distinguish viable tumour from scar tissue by CT or MRI [12].

Molecular Nuclear Medicine imaging techniques—Single Photon Emission Computed Tomography (SPECT) and Positron Emission Tomography (PET)—although not routinely used yet, could provide complementary information to CT and MRI, to establish a noninvasive histological diagnosis prior to operation, to distinguish between residual or recurrent viable tumour and scar tissue and to estimate treatment response in the postsurgical and postradiation evaluation. In this purpose, various SPECT and PET radiopharmaceuticals have been used which provide information about various cellular processes and biological characteristics of meningiomas (Table 1).

## 2. SPECT Radiopharmaceuticals

### 2.1. Thallium-201

Thallium-201 (^201^Tl) is a potassium analogue possessing an affinity for the sodium- and potassium-activated adenosine triphosphatase (Na^+^-K^+^ ATPase) pump and is distributed in potassium-rich organs, such as the heart, kidney, gastrointestinal tract, and the thyroid gland, but exhibits little uptake in the normal brain [13].

^201^Tl is known to accumulate in a variety of tumors, and it was one of the first radiotracers that have been used in the area of oncology. ^201^Tl uptake in brain tumors depends on regional blood flow, Blood Brain Barrier (BBB) permeability, cellular activity, and cell number [14].

^201^Tl uptake in meningiomas was thought mainly to be related to lesional vascularity. Nevertheless, its retention rates differed according to histological types indicating that vascularity itself would not correlate with histological type or malignancy grade, and factors other than lesional vascularity were involved. Mitotic or proliferation rates correlated to cellular Na^+^-K^+^ ATPase activity would be a factor regulating ^201^Tl uptake. It has been reported that long-lasting retention of thallium in serial brain SPECT studies could differentiate preoperatively intracranial meningiomas with different biological behaviour, predict malignant potential of meningiomas, and provide information of meningioma aggressiveness [15–17]. In a recent study by Takeda et al., a significant correlation was found between the ^201^Tl uptake index in the delayed image and MIB 1 labeling index in postoperative tumor specimens. Moreover, meningiomas with strongly positive vascular endothelial growth factor (VEGF) exhibited a significantly higher ^201^Tl uptake index compared to VEGF weakly positive meningiomas in both the early image and the delayed image [18].

### 2.2. ^99m^Technetium-Labelled Compounds

^99m^Technetium-(^99m^Tc-) labelled compounds have also been used in the study of meningiomas. These were proved advantageous over ^201^Tl, due to 140 keV *γ*-ray energy, high photon flux, higher spatial resolution, less radiation burden to the patient, and excellent availability.

^99m^Tc-methoxyisobutylisonitrile (MIBI) is a common radioligand for the evaluation of myocardial perfusion. SPECT with ^99m^Tc-MIBI has been proposed as a complementary diagnostic tool for the evaluation of various neoplasms including brain tumours [19]. The cellular uptake of positively charged MIBI ions is driven by the negative electric potential generated on the inner surface of the cellular membrane and particularly on the inner mitochondrial membrane [20]. ^99m^Tc-MIBI uptake is related to tissue perfusion and normal cell membrane and mitochondrial activity. It has been described by Petrovic et al. [21] that meningiomas exhibit high perfusion index but low retention index. This phenomenon was attributed to a relatively weak fixation of the radiopharmaceutical in benign meningioma cells. The high metabolic rate of malignant cells generates very high negative electrical potential along the inner surface of their mitochondrial membranes that strongly attracts MIBI cations. As the metabolic demands of meningioma cells are much lower than those of malignant tumours, the fraction of MIBI ions stranded in the mitochondria is smaller than in malignant cells, and the larger fraction is available to the washout mechanism. Hyperperfusion of meningiomas can also facilitate the washout. Thus, it seems likely that both the high uptake and the quick wash-out of MIBI observed in meningioma are facilitated by the abundant blood supply and the lack of BBB. Bagni et al. [19] also noticed that ^99m^Tc-MIBI uptake in meningiomas was proportional to the tumour vascularity. It has also been shown that high-grade brain tumors have increased ^99m^Tc-MIBI uptake compared with that of low-grade brain tumors. ^99m^Tc-MIBI uptake was correlated with the percentage of the S-phase fraction of the tumor cell (which is a reflection of the proliferation potential of tumors) and the DNA aneuploidy level (which is more common in malignant tumors). The inactive, nonviable, apoptotic, or necrotic tumour cells do not take up the radioligand and that is why it is sometimes referred to as the viability marker [22].

In malignant tumors the uptake of ^99m^Tc-MIBI has also been associated with the presence of P-glycoprotein (Pgp) in the cell membrane which is related to the multidrug resistant gene [23]. Kunishio et al. evaluated whether ^99m^Tc-MIBI SPECT characteristics of intracranial meningioma were correlated with the histological malignancy, proliferative potential, and Pgp expression, encoded by the multidrug resistance gene-1 (*MDR-1*) messenger ribonucleic acid (mRNA). They found that ^99m^Tc-MIBI may not be useful for determining proliferative potential and histological malignancy, but could predict anticancer drug resistance related to the expression of *MDR-1* mRNA and its gene product Pgp in patients with intracranial meningiomas [24].

^99m^Technetium—tetrofosmin (^99m^Tc-TF)—a lipophilic cationic diphosphine, which is routinely used for myocardial perfusion imaging, displays tumor-seeking properties, too. Its uptake mechanism is similar to ^99m^Tc-MIBI, depending mainly on regional blood flow and cell membrane integrity. ^99m^Tc-TF enters cells mainly via passive transport driven by the negative potential of the intact cell membrane and localizes mostly within the cytosol and only a fraction enters the mitochondria [25]. In the healthy brain, uptake of ^99m^Tc-TF is seen in the choroid plexuses, pituitary gland, and scalp, but not in the normal brain parenchyma, since it does not cross the intact BBB [26].

**Figure 1:**
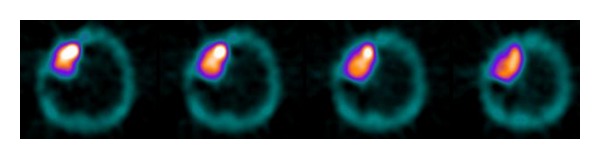
Transverse slices of a ^99m^Tc-tetrofosmin SPECT brain study in a patient with meningioma showing increased tracer uptake by the tumor.

In a small series of studies, ^99m^Tc-TF uptake in meningiomas was correlated with cellular proliferative activity (assessed either immunohistologically by the Ki-67 index or by flow cytometry) and tumor grading (Figure 1) [27, 28]. A significant correlation of  ^99m^Tc-TF uptake with proliferation index and tumor grade was found. Anaplastic meningiomas tended to exhibit a higher radiotracer uptake as compared to typical ones without any overlapping of  ^99m^Tc-TF uptake between them. Likewise, meningiomas with higher proliferation index tended to exhibit increased ^99m^Tc-TF uptake. Furthermore, it was also found a significant correlation of ^99m^Tc-TF uptake with risk of recurrence at 1 year postoperatively. Thus the authors stated that ^99m^Tc-TF brain SPECT may hold a role in distinguishing typical from anaplastic meningiomas preoperatively, a discrimination that is difficult by CT or classic MRI, and that this finding would also be of great importance in patients in which surgery is not an option, due to tumor localization, or when there is a medical disorder that could increase the potential morbidity of surgical excision. Nevertheless, a limitation of these studies was the absence of grade II meningiomas in the study population. 

### 2.3. Somatostatin Receptor Scintigraphy

Somatostatin—a cyclic tetradecapeptide neuropeptide—is the most widely distributed of the hypothalamic releasing hormones in the central nervous system and in the periphery, including the pancreas, gut, and pituitary [29]. In the brain, somatostatin is believed to act as a neurotransmitter and neuromodulator [30]. The effects of somatostatin are mediated by transmembrane domain G-protein coupled receptors. In vivo and in vitro studies have shown that somatostatin receptors (SSTRs) are expressed on the cell membrane of various central nervous and peripheral tissues in high density to a varying extent, including tumours of neuroendocrine origin and intracranial tumours [31]. Molecular biologic research revealed that various types of SSTRs exist. To date, 5 different SSTRs subtypes are known, SSTR1–SSTR5, with subtype specificity for distinct histology [32, 33]. Of these, subtype 2, SSTR2, is most often expressed on the surface [34–36]. Two isoforms of SSTR2 have been isolated, SSTR2A and SSTR2B, which differ in size and the sequence of their intracellular COOH-terminal domain [37].

It is now well known that leptomeninx express SSTRs [38]. Meningiomas, which originate from the arachnoid layer of the leptomeninx, usually have a high SSTR2 density [39].

Clinical nuclear medicine has taken advantage of this characteristic, making the in vivo detection of SSTRs possible. Somatostatin receptor scintigraphy (SSRS) became an invaluable tool used extensively in routine management of patients with meningiomas.

As endogenous somatostatin has a very short biologic half-life (*∼*2 min), it is rapidly cleared from the blood; thus, somatostatin itself cannot be used as an imaging agent in nuclear medicine. To overcome this drawback, a long-acting analog of somatostatin consisting of 8 amino acids was developed. This octapeptide exhibits a biologic half-life on the order of several hours and can be linked through diethylenetriaminepentaacetic acid to indium-111 (^111^In), forming the well-known radiotracer [^111^In]octreotide [40].

Although all receptors bind natural somatostatin with high affinity; nevertheless, they differ in their binding characteristics to long-acting somatostatin analogues. Whereas SSTR2, SSTR3, and SSTR5 exhibit high affinity for octreotide [41], the frequent overexpression of the SSTR2A receptor may explain the high tracer uptake observed in meningioma patients during SSRS [42].

SSRS does not reveal increased tracer uptake by the normal leptomeninx, although the latter contains SSTRs. Since octreotide is a polar, water-soluble peptide that does not penetrate the intact BBB, SSRS can only demonstrate somatostatin receptor positive intracranial lesions if the BBB is disrupted. Only meningiomas—located outside the BBB—usually demonstrate high tracer uptake, while intracranial tumours may be out of reach due to the BBB and therefore may not be detectable by SSRS [43, 44].

Meningiomas visualization with ^111^In-octreotide depends on the expression of SSTR2 on meningioma cells—as shown by the reverse transcriptase polymerase chain reaction—[45], as well as on the tumor volume [46].

**Figure 2:**
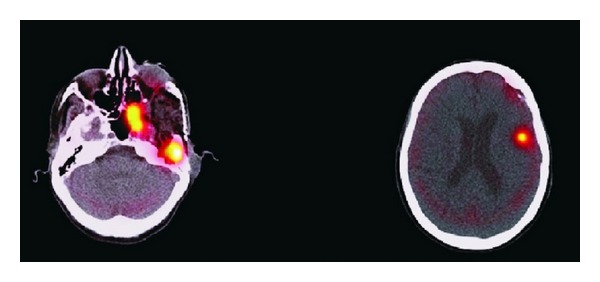
Fused SPECT/CT images of somatostatin receptor scintigraphy demonstrating increased tracer uptake in a patient with anaplastic meningioma [67].

Although many studies have reported detection of all meningioma lesions with a sensitivity almost 100% [47–50] and varied ^111^In-octreotide uptake (Figure 2); however, a major drawback of ^111^In-octreotide SPECT is its difficulty in detecting meningiomas with a diameter <2.7 cm or a volume <10 mL [51]. A significant correlation between meningioma volume and grade of tracer uptake was found, with higher uptake grade in larger tumours. Howbeit, depending on location and receptor status, even small tumours with volumes of 5 mL demonstrated intense tracer uptake [46]. Despite the high sensitivity of ^111^In-octreotide scintigraphy for the diagnosis of meningiomas, specificity may be low depending on the presence of lesions which disrupt the BBB, such as cerebral metastases [44]. Nevertheless, the method provides high negative predictive value (100%).

Due to the high SSTRs density, meningiomas of the skull base or the orbit may be differentiated from somatostatin receptor-negative tumours using ^111^In-octreotide scintigraphy. Neurinomas and neurofibromas do not express somatostatin receptors, thus they do not demonstrate tracer uptake, making ^111^In-octreotide scintigraphy a useful technique in the differential diagnosis from multiple meningiomas [52, 53].

SSRS seems to be an additional valuable tool with high sensitivity and specificity for optimizing the diagnosis of optic nerve sheath meningiomas from other orbital tumors (such as vascular lesions, non-hodgkin lymphomas, optic nerve gliomas, idiopathic orbital inflammation), without the need for more invasive procedures. It has been reported that the ^111^In-octreotide uptake ratios of meningiomas were significantly higher than those of other subgroups of orbital tumors, except for adenocarcinomas [54].

SSRS may also aid in the differentiation between postoperative scar and meningioma recurrence. The risk of local recurrence is wellknown, especially for meningioma located near the skull base where meninges are attached very closely to the bones, thus rendering total resection of meningioma difficult. In the first 6 months after surgery, in a reasonable number of patients, MRI may fail to differentiate between tumor remnants or recurrent meningioma and nonspecific hyperperfusion. The high tumour ^111^In-octreotide uptake seen in SSRS in most meningiomas may be of clinical relevance for exact tumour delineation in cases of tumour recurrence [55–58].

It has been reported that the correlation between SSTR2A immunoexpression and the histological grade, according to the WHO 2007 classification, as well as microvessel density of the tumors, has demonstrated that atypical and anaplastic meningiomas characterized by a higher microvessel density. This finding would provide the basis for the use of somatostatin analogue-based therapies, which have an antiangiogenic effect, in the treatment of these tumours [59]. Nevertheless, in a small number of pathologically proven meningiomas, Nathoo et al. reported that there was no difference in intensity of octreotide uptake related to the histologic subtype of meningiomas [44].

The limited imaging properties using a medium-energy isotope, such as ^111^In, explain the reduced sensitivity in detecting meningiomas with a volume of less than 10 mL so that SSTRs imaging with higher resolution is desirable. A new ^99m^Tc-labeled somatostatin analog, ^99m^Tc-depreotide, was developed and approved in the United States and Europe for diagnostic use in lung nodules [60].

The binding profile of this new tracer shows high affinity to subtypes 2, 3, and 5 of the SSTRs, with dissociation constants (*K*_d_) in the order of 1.5 to 2.5 nmol/L [61]. Therefore, it is similar to ^111^In-octreotide, which binds to SSTRs subtypes 2 and 5 with high affinity (*K*_d_, 0.1 to 5 nmol/L), to subtype 3 with moderate affinity (*K*_d_, 10 to 100 nmol/L), but does not bind to SSTR subtypes 1 and 4 [62]. Compared with ^111^In-octreotide, the use of ^99m^Tc-depreotide has the advantage of a lower radiation dose due to the faster decay and lower photon energy of the isotope, resulting in an effective dose estimate of 0.023 mSv/MBq [63] instead of 0.08 mSv/MBq for ^111^In [64]. Furthermore, the cost of the ^99m^Tc-labeled radiopharmaceutical is less and there are fewer requirements for the imaging equipment and the radiation protection facilities compared with those required when the octreotide analog labeled with the medium-energy isotope ^111^In is used. Labeling of the ligand is easy and generator produced ^99m^Tc ensures its continuous availability. 

^99m^Tc-depreotide SPECT has been used for the evaluation of intraorbital and extracranial recurrent meningioma in comparison with ^111^In-octreotide and revealed a smaller extracranial site of recurrence, not detected on ^111^In-octreotide scintigraphy, as a result of the better resolution that can be achieved with that isotope [65].

## 3. PET Radiopharmaceuticals

### 3.1. ^18^F-2-Fluoro-2-deoxy-D-glucose

**Figure 3:**
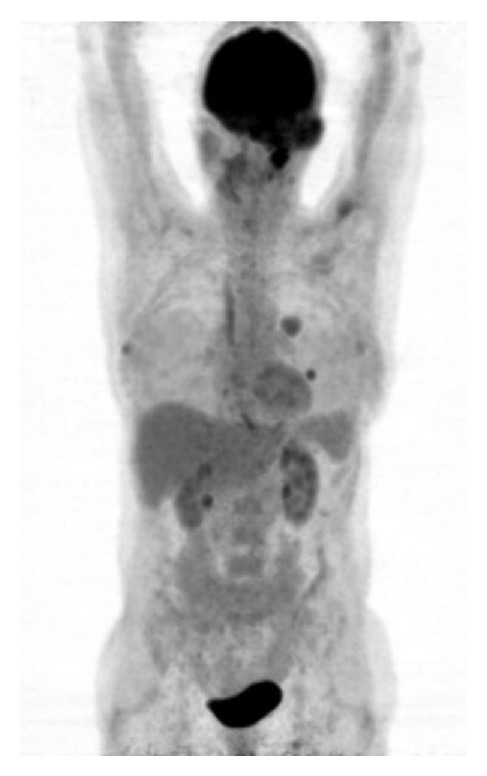
^18^F-FDG PET study in a patient with anaplastic meningioma. Increased FDG uptake in the primary tumor (left temporal region) as well as in its pulmonary metastases [67].

^18^F-2-fluoro-2-deoxy-D-glucose (^18^F-FDG) is used for the study of brain glucose metabolism. It is transported into the cells by facilitated diffusion, then phosphorylated to FDG-6-PO_4_ and trapped intracellularly where it can be measured. ^18^F-FDG was one of the first PET tracers used in the study of meningiomas although with conflicting findings in the literature. Some authors have described high ^18^F-FDG uptake in meningiomas, similar to normal gray matter, whereas others reported hypometabolic features resulting in low tumor-to-gray matter-ratio [66–68]. It has been proposed that these differences may be attributed to differences in glucose consumption in tumors with different biological features (Figure 3). ^18^F-FDG uptake was proposed as an index of tumor aggressiveness and it has been correlated with the probability of recurrence and patients' survival [66, 69, 70]. It has been reported that the highest glucose utilization rate among intracranial meningiomas was seen in atypical, papillary and recurrent meningiomas [44, 66] or in grade 2 and 3 meningiomas (according to previous WHO classification) as compared with grade 1 meningiomas in tumors of high cellularity [69]. Especially for WHO Grade 1 meningioma ^18^F-FDG PET seems not useful for tumor delineation, which might be further troubled by peritumoral edema [66, 70, 71]. Cremerius et al. reported that recurrent meningioma exhibited more intense FDG uptake than grade 1 meningioma as compared with gray matter [70]. In a recent study, Liu et al. found ^18^F-FDG PET neither useful for exact tumor delineation nor for monitoring response to radiosurgery [72]. However, they described that ^18^F-FDG may be valuable for differentiating benign from malignant meningioma. In contrast, Lee and coworkers stated that the sensitivity of FDG PET to detect high-grade meningioma was low, but ^18^F-FDG uptake correlated significantly with the proliferative activity of meningiomas. The authors suggested that ^18^F-FDG uptake could be one of the significant prognostic factors and that its role would rather be to predict recurrence and survival [68].

Nevertheless, the major drawback of ^18^F-FDG is its high uptake in normal gray matter. Thus, in slow growing tumors such as meningiomas—which may exhibit a moderately increased glucose metabolism—^18^F-FDG PET may not reliably detect them. Furthermore, ^18^F-FDG is not tumor specific, since high uptake has also been observed in infection or inflammation [73]. Therefore, other tracers have been tested.

## 4. PET Tracers Beyond FDG

### 4.1. Amino Acids

The rationale to use amino acids in proliferating cells is based on an upregulation of amino acid transport resulting in a high tumor to normal tissue ratio—even in slow growing tumors [74, 75]. Several labelled amino acids have been used in the study of meningiomas such as [^11^C]-methionine (^11^C-MET) and [2-^ 18^F]-fluoro-L-tyrosine (^18^F-TYR).

^11^C-MET is a marker of amino acid transport as well as protein synthesis and shows high uptake in meningioma (tumour to cerebellum ratios 2.62–5.37, mean 3.63) [67, 76–78]. As there is a comparatively low uptake in normal white and gray matter, it allows better delineation of meningioma from normal structures than ^18^FDG [79]. Astner et al. reported its use in planning stereotactic radiotherapy of skull base WHO I and WHO II meningiomas [80]. They found that the addition of ^11^C-MET PET in the delineation of Gross Tumor Volume (GTV) resulted in an enlargement of GTV of 9.4% ± 10.7% since the method detected small tumor portions that were not identified by CT or MRI. Thus they concluded that the integration of the method in radiotherapy treatment planning could be beneficial for the management of patients. It has been reported that ^11^C-MET uptake is not correlated with aggressiveness and WHO grading of meningiomas [79, 81], although Iuchi et al. found that ^11^C-MET uptake significantly correlated with proliferative tumor activity (Ki-67 index) [82].

^18^F-TYR is another marker of L-amino acid transport protein synthesis and accumulates significantly in meningioma (tumor-to-cortex activity ratio of about 2.5). The uptake in normal gray and white matter is low, but high in oropharyngeal mucosa and salivary glands. ^18^F-TYR was used for the discrimination of meningioma from healthy tissue, especially in difficult locations such as the skull base, and the delineation of tumor compared to that seen on MRI [83]. It was found that ^18^F-TYR PET could clearly identify meningiomas of the base of the skull even after irradiation, while the metabolic lesions were not exactly matched with the MRI findings. The clinical significance of this finding should be clarified in further studies in order to define the role of ^18^F-TYR in treatment planning.

### 4.2. ^11^C-Choline

^11^C-Choline is a marker of phospholipid synthesis, which is increased in malignant tumors. It has been used for imaging of various tumors including prostate cancer and brain tumors [84, 85]. The uptake of ^11^C-choline in the normal brain is negligible, allowing good delineation of brain tumor contours and diagnosis of tumor recurrence [85]. Meningiomas have exhibited, in in vitro and imaging studies, altered choline metabolism [86]. Preliminary results of the use of ^11^C-choline PET in meningiomas demonstrated excellent visualization of all lesions [87, 88]. Giovacchini et al. [88] showed in a preliminary clinical study including 7 meningioma patients (WHO I = 5, WHO II = 2) that ^11^C-choline may better image meningioma compared to FDG. Furthermore, it was found that ^11^C-choline uptake was higher in grade II than in grade I meningiomas, indicating that ^11^C-choline uptake may reflect the proliferation rate of the tumors and it could be used for meningioma grading. However, the potential of this tracer remains unclear and further studies are needed to confirm these findings as well as to assess the clinical use of the tracer in the management of meningioma.

### 4.3. 1-^11^C-Acetate

1-^11^C-Acetate has been used for the detection of several tumors such as prostate and lung cancer, hepatocellular carcinoma and astrocytomas [89–92]. The possible mechanisms of 1-^11^C-acetate uptake by tumor cells include impaired tricarboxylic acid cycle activity or anabolic pathways of fatty acid and sterol synthesis [93, 94]. In the brain, acetate is preferentially metabolized by astrocytes, and labeled acetate has been used to assess glial metabolism and glial-neuronal interactions [95]. Metabolic data derived from a small set of tumors revealed that glioblastoma and meningioma clearly can convert ^14^C-acetate into acidic and amino acid metabolites, presumably through the oxidative pathway of the tricarboxylic acid cycle. In a study by Liu et al., it was found that 1-^11^C-acetate was useful for detecting and evaluating the extent of meningioma and potentially useful for monitoring the response to radiosurgery. The authors suggested that accurate tumor delineation with 1-^11^C-acetate PET is of potential value for guiding the stereotactic biopsy and for optimizing treatment planning before radiosurgery. However, they found that 1-^11^C-acetate was not helpful for grading the tumor [72].

### 4.4. ^13^N-NH_3_ PET

There are 2 chemical forms of ammonia (NH_3_ and NH_4_^+^) in the blood. The relative proportion of NH_3_ and NH_4_^+^ in blood is determined by the pH. The majority of blood ammonia is in the form of NH_4_^+^ at a normal pH of 7.3 to 7.4. Similar to Tl-201, (13)N-(13)NH(4)(+) is an analog of K(+) and could substitute K(+) in some cases because they have similar hydrated radius [96]. It has been reported that cerebral astrocytomas exhibit a substantial uptake of ^13^N-NH_3_ and ^13^N-NH_3_ might be a promising tracer for separating radiation necrosis from astrocytoma recurrence [97, 98]. Recently, ^13^N-NH_3_ has been used in the study of meningiomas by Xiangsong et al. [99]. These preliminary results indicate that ^13^N-NH_3_ allows better detection and evaluation of the extent of meningiomas in comparison with FDG, offering excellent imaging properties and a very high tumor-to-background ratio. The brain uptake of ^13^N-NH_3_ is limited due to its low lipid solubility which does not permit passage through the intact BBB. The location of meningiomas outside the BBB results in higher accumulation of the tracer in these tumors than in the normal brain cortex. Nevertheless, ^13^N-NH_3_ uptake was not useful for differentiating benign (Grade I) from atypical (Grade II) meningiomas.

### 4.5. ^68^Ga-DOTATOC

The last decade, the somatostatin analog DOTA-D-Phe1-Tyr3-octreotide (DOTA-TOC) has been developed [100]. 1,4,7,10-tetraazacyclododecane-*N,N*9*,N*0*,N*-tetraacetic acid (DOTA) is a macrocyclic chelator that ensures high in vivo stability for the corresponding radiometal chelates. Moreover, the replacement of Phe3 by Tyr in the octapeptide increases its hydrophilicity and thus the efficiency of its clearance by the kidney. DOTA-TOC has been labelled with the positron emitter ^68^Ga. This new radiotracer, ^68^Ga-DOTATOC, shows a high affinity (half-maximal inhibitory concentration [IC_50_] =  14 nmol/L) for human SSTR2 [101] and exhibits several advantages over conventional ^111^In-octreotide [102]. The coincidence detection of two photons generated by annihilation of the emitted positron by a modern PET scanner facilitates a geometric resolution in the order of 4–6 mm, and biodistribution can be quantified in (patho)physiologic terms. Because of the increased spatial resolution and the ability to quantify biodistribution, PET is desirable for SSTR imaging.

**Figure 4:**
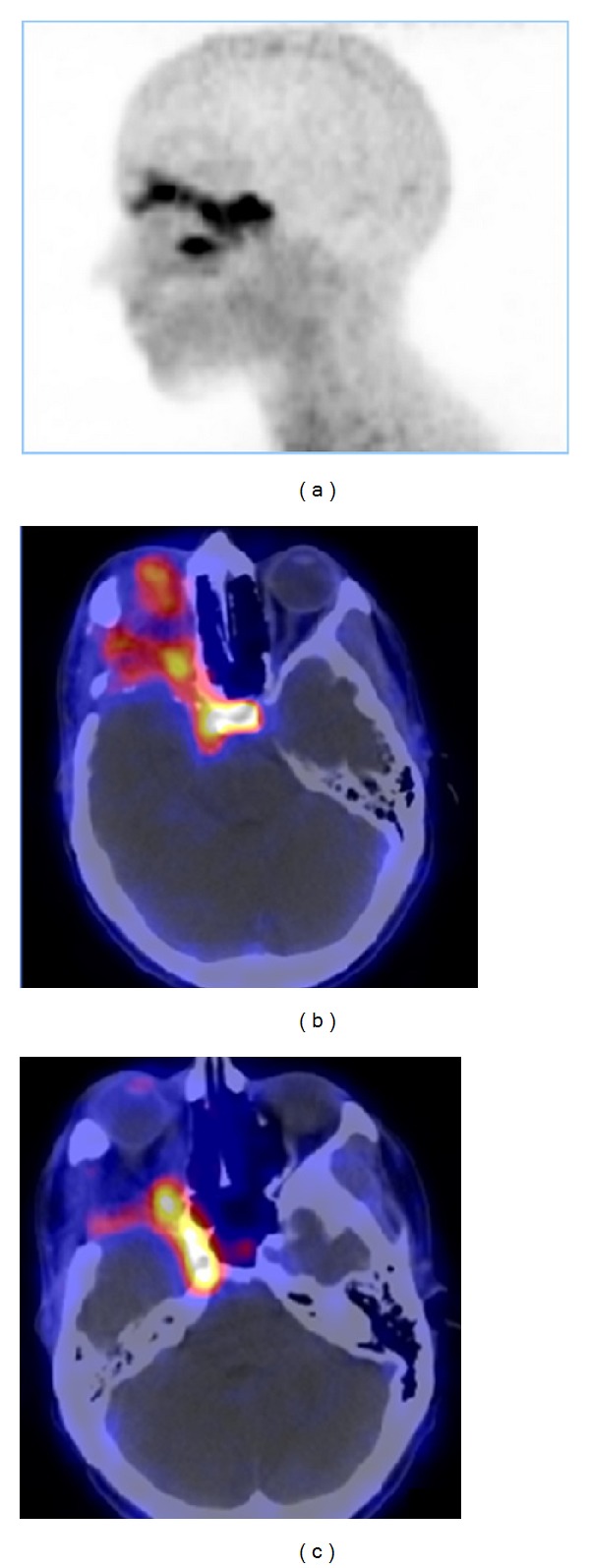
^68^Ga-DOTATOC PET (a) and fused PET/CT (b and c) images of a skull base meningioma with orbital invasion and close relation to the sella turcica region. Physiological tracer uptake of the pituitary gland [106].

^68^Ga-DOTATOC PET showed a high meningioma/background ratio [102, 103]. Henze et al. [102, 103] reported high-contrast images of meningiomas measuring only 7-8 mm in diameter, which could be clearly separated both from surrounding brain and bone tissue. This capability is important since meningiomas may cause serious problems to the neurosurgeon because of their tendency to local osseous invasiveness. Especially at the skull base, where biopsy has a high risk of hemorrhage and the findings of CT and MRI are often unclear [9, 104], PET provides valuable additional information regarding the extent of meningiomas located beneath osseous structures. A similar difficulty arises with *en plaque *meningioma [105], where the surgeon has to limit the resection to a reasonable degree. Therefore, preoperative evaluation of meningioma with ^68^Ga-DOTATOC PET, or more ideally, fused PET/CT or PET/MRI imaging, would influence surgical strategy and increase the accuracy of surgical resection. However, one limitation is the parasellar region, because of the pituitary gland expressing SSTR2 (Figure 4) [106, 107]. ^68^Ga-DOTATOC also allows detection of additional lesions in patients with multiple meningiomas [107].

The ability of ^68^Ga-DOTATOC for adequate delineation of meningiomas has proven useful for radiation treatment planning of these tumors [106–109]. It has been reported that ^68^Ga-DOTATOC-PET/CT information may strongly complement anatomical data from MRI and CT and improve target volume definition especially in cases with complex meningioma such as skull base manifestations, osseous infiltration, and recurrent disease after surgery. Radiation targeting with fused ^68^Ga-DOTATOC PET, CT, and MRI resulted in significant alterations in target definition in 73% of patients [109].

^68^Ga-DOTATOC may be used for the evaluation of alternative therapeutic approaches in cases of nonoperable meningiomas. Pretherapeutic knowledge of the receptor status is essential for both, either the application of hormonal treatment or the use of DOTATOC labelled with *β*-emitting radionuclides, a field of nuclear medicine in which interest has been growing rapidly in the past few years [110, 111].

## 5. Conclusion

The contribution of SPECT and PET molecular imaging in the noninvasive diagnosis and differential diagnosis, response to treatment, and target delineation for radiation therapy of meningioma, is well described. SPECT and PET may also help in the detection of early metabolic changes, which precede anatomical changes revealed by CT and MRI, and identify metabolically active and aggressive tumor areas, which should be treated appropriately.

## Figures and Tables

**Table 1:** SPECT and PET radiopharmaceuticals used in meningiomas.

Tracer	Imaging modality	Advantages	Disadvantages	References
Thallium-201	SPECT, analog of K^+^	Information of tumor biological characteristics	Limited imaging properties, serial brain SPECT studies	[15–18]

^99m^Tc-labeled compounds	SPECT, tissue perfusion, cell membrane integrity, and mitochondrial activity	Viability marker, prediction of anticancer drug resistance related to Pgp	Small series of patients, the correlation between tracer uptake and tumor grading or other biological characteristics, needs validation with further studies	[19, 21–28]

^111^In-octreotide and ^99m^Tc-depreotide	SPECT, SSTR	High sensitivity and negative predictive value, differential diagnosis from somatostatin receptor-negative and orbital tumours, differentiation between postoperative scar and recurrence, selection of patients for somatostatin analogue-based therapies	Specificity depends on the BBB integrity, difficulty in detecting small tumors, limited imaging properties of ^111^In, few studies with ^99m^Tc-depreotide	[42–59, 65]

^18^F-FDG	PET, brain glucose metabolism	Prognostic information (prediction of recurrence and survival)	High uptake in normal gray matter not tumor specific	[44, 66–73]

^11^C or ^18^F labeled amino acids	PET, protein synthesis	High tumor/background ratio, identification of skull base meningiomas, improve target volume definition for RT	Not useful for grading	[67, 76–83]

^11^C-choline	PET, phospholipid synthesis	Meningioma grading	Few studies	[87, 88]

1-^11^C-acetate	PET	Accurate tumor delineation, guiding the stereotactic biopsy, optimizing treatment planning before radiosurgery	Not useful for grading, few studies	[72]

^13^N-NH_3_	PET, analog of K^+^	High tumor/background ratio	Not useful for grading, Few studies	[99]

^68^Ga-DOTATOC	PET, SSTR	High tumor/background ratio, identification of skull base and en plaque meningioma and local osseous invasiveness, improvement of target volume definition for RT, recurrent disease, selection of patients for hormonal treatment or the use of DOTATOC labelled with *β*-emitting radionuclides	Uptake in parasellar lesions	[102, 103, 105–111]

Pgp: P-glycoprotein, SSTR: somatostatin receptors, BBB: Blood Brain Barrier, RT: Radiation Treatment.
